# Immunogenicity of *Theileria parva* p67C Antigen Delivered via Adjuvanted CoPoP Liposomes in Cattle and Mice

**DOI:** 10.3390/vaccines14050459

**Published:** 2026-05-20

**Authors:** Harriet Oboge, Wei-Chiao Huang, Gabriel Aboge, Hannah Chege, Rose Ojuok, Naomi Chege, Joel Musando, Elizabeth Jane Poole, Samuel Mwangi Thumbi, Vishvanath Nene, Jonathan F. Lovell, Anna Lacasta

**Affiliations:** 1 Health Program, International Livestock Research Institute, Nairobi P.O. Box 30709, Kenya; obogeharriet@gmail.com (H.O.); wambuiche08@gmail.com (H.C.); r.ojuok@cgiar.org (R.O.); naomi.chege@ucalgary.ca (N.C.); v.nene@cgiar.org (V.N.); 2 Department of Public Health Pharmacology and Toxicology, Faculty of Veterinary Medicine, University of Nairobi, Nairobi P.O. Box 30197, Kenya; gaboge@uonbi.ac.ke; 3 Department of Biomedical Engineering, University of Buffalo, Buffalo, NY 14260, USA; weichiao@buffalo.edu (W.-C.H.); jflovell@buffalo.edu (J.F.L.); 4 Department of Veterinary Pathology, Parasitology and Microbiology, Faculty of Veterinary Medicine, University of Nairobi, Nairobi P.O. Box 30197, Kenya; 5 Department of Biological Sciences, Faculty of Science, University of Calgary, Calgary, AB T2N 1N4, Canada; 6 Host Parasite Interactions Training Network, University of Calgary, Calgary, AB T2N 1N4, Canada; 7 KAVI Institute of Clinical Research, University of Nairobi, Nairobi P.O. Box 19676, Kenya; jmusando@uonbi.ac.ke; 8 Data and Research Methods Unit, International Livestock Research Institute, Nairobi P.O. Box 30709, Kenya; j.poole@cgiar.org; 9 Institute of Immunology and Infection Research, School of Biological Sciences, University of Edinburgh, Edinburgh EH8 9YL, UK; thumbi.mwangi@wsu.edu; 10 Paul G. Allen School for Global Health, Washington State University, Pullman, WA 99164, USA; 11 Centre for Epidemiological Modelling and Analysis, University of Nairobi, Nairobi P.O. Box 30197, Kenya

**Keywords:** *Theileria parva*, ECF, p67C antigen, nanoparticles, CoPoP liposomes, adjuvants, cattle, mice

## Abstract

**Background:** Effective vaccines are essential to overcome the limitations of livestock immunisation, particularly in low- and middle-income countries (LMICs), where scalable, thermostable, and easy-to-administer solutions are needed. Nanoparticle-based delivery systems, such as the Spontaneous Nanoliposome Antigen Particle (SNAP) technology using CoPoP liposomes, offer a promising alternative for subunit vaccine development, although their performance in large animal species remains poorly characterised. CoPoP enables the rapid non-covalent multimeric display of His-tagged protein antigens combined with immunomodulators on liposomes incorporating cobalt porphyrin–phospholipid (CoPoP). **Objective:** To evaluate the immunogenicity of CoPoP-based liposomes delivering the *Theileria parva* p67C antigen in cattle and compare their performance in murine models. **Methods:** Cattle and mice were immunised with p67C formulated in CoPoP liposomes incorporating QS-21 and/or PHAD immunomodulators. Humoral and cellular responses were assessed. Parallel in vitro stimulation of bovine PBMC with Quil-A was used to investigate the mechanistic effects of saponins on bovine cells. **Results:** CoPoP liposome formulations did not improve p67C immunogenicity in cattle, with antibody responses at least two-fold lower than previously reported results and no detectable cellular responses. In contrast, the same platform induced up to 2000-fold higher antibody titres in mice. This disparity is likely driven by differences in antigen dose relative to body mass, tissue architecture, lymphatic accessibility, and innate immune signalling differences. PHAD-mediated TLR4 activation appeared less effective in cattle, whereas QS-21 induced a broader immune activation, likely through conserved inflammasome pathways. Despite limited immunogenicity, antigen presentation by CoPoP liposomes was preserved. **Conclusions:** SNAP-based CoPoP liposomes showed strong immunogenicity in mice but limited efficacy in cattle, highlighting the challenges of cross-species translation. Optimisation of antigen dose and adjuvant selection for the targeted species is required, with QS-21 representing a more promising candidate than the TLR4 agonist. The scalability and versatility of SNAP technology support its continued development for multivalent livestock vaccines.

## 1. Introduction

The field of livestock vaccinology is evolving rapidly to address the unique challenges of veterinary vaccine development, such as the diversity of target species, the need for scalable and cost-effective delivery systems, and deployment in low- and middle-income countries (LMICs). These regions in particular demand thermostable, small-dose, accessible, and easy-to-administer vaccines to meet the needs of smallholder farmers [1,2,3].

To overcome these barriers, researchers are increasingly turning to integrated scientific approaches, such as “systems vaccinology,” an interdisciplinary field that merges transcriptomics, proteomics, immunology, computational biology, structural biology, and bioinformatics, among others [4,5]. This holistic framework enables a more efficient identification of promising vaccine candidates and immune correlates of protection. Interdisciplinary approaches have made technologies that were once considered unattainable, such as mRNA or saRNA vaccines, readily available for broad applications, including in livestock [6]. A simple, potent, robust, safe, and adaptable vaccine platform could dramatically accelerate candidate vaccine evaluation and broaden access, especially for neglected livestock diseases affecting resource-limited settings [7,8,9].

Nanotechnology provides innovative approaches with great potential to enhance vaccine efficacy. The use of nanoparticles in vaccine delivery is an effective strategy owing to their small size, usually below 1 μm in diameter, making them efficiently phagocytosed by the antigen-presenting cells (APCs) [10]. The increase in antigen uptake results in increased antigen presentation to the immune system [11,12]. In addition, nanoparticles promote preservation of antigen structure and controlled antigen release, which prolongs the immune system stimulation [10]. Taking all these characteristics together, the use of nanoparticles enhances the immunogenicity of antigens.

Nanoparticles explored for use in veterinary vaccines include liposomes, polymers, and virus-like particles (VLPs), all of which can be engineered to facilitate efficient antigen presentation to the immune cells [13,14]. Some examples of improvements are the co-delivery of the nanoparticle–antigen with adjuvant molecules, enhancing the immune response even further by targeting specific cells and pathways, and the incorporation of metals such as cobalt and nickel, which enhance antigen binding and presentation [15].

The Spontaneous Nanoliposome Antigen Particleisation (SNAP) vaccine platform uses liposomes incorporating metal-chelating lipids to rapidly capture and display His-tagged proteins in a multimeric form. The technology offers a rapid plug-and-play system for the delivery of multimerised antigens, an alternative to less plastic systems, such as virus-like particles (VLPs), ferritin, or outer membrane vesicles (OMVs) [16]. The technology enables particle formation without the need for chemical conjugation or deep knowledge of the protein/antigen structure [15]. Another advantage is that the technology allows the incorporation of immunomodulators in the same liposome where the antigens are displayed, and the nanoliposomes can be lyophilised to create thermotolerant formulations [15]. Remarkably, CoPoP liposomes have been demonstrated to be very stable after injection [17] and to have an extended shelf life after lyophilisation [18].

In a recent study, the use of monophosphoryl lipid A (MPLA, a TLR4 agonist) and QS-21 (a saponin) co-formulated on SNAP liposomes containing cobalt porphyrin–phospholipid (SNAP/CoPoP) with a stabilised Pfs48/25-derived *Plasmodium falciparum* protein as a model antigen elicited 50-fold higher antibody titres than the administration of adjuvanted soluble protein in mice [19]. Other studies also support the potency and flexibility of the SNAP platforms. Ten recombinant surface hemagglutinin antigens representing distinct influenza virus strains generated highly multivalent nanoparticles with good antibody responses in mice and ferrets to each antigen [20]. The SNAP/CoPoP has entered human clinical trials as a component of a COVID-19 vaccine, EuCorVac-19 (ECV-19, NCT05603052). This SARS-CoV-2 receptor-binding domain (RBD)-based vaccine, formulated with CoPoP liposomes containing MPLA, has completed its Phase III clinical trial and demonstrated great promise [21]. However, the potential of this technology in large livestock animals remains to be evaluated.

A good model antigen to evaluate the capacity of the SNAP technology to help stimulate an immune response in large animals is p67, which is the major surface antigen of the sporozoite stage of the *Theileria parva* parasite, the causative agent of East Coast fever (ECF), a devastating disease affecting cattle in sub-Saharan Africa. p67 is the target of sporozoite-neutralising antibodies and can induce immunity to ECF. Soluble p67 and various sub-fragments, including the 80 amino acid C-terminal section, p67C, have been extensively used in experimental vaccines (reviewed in [22]). Since p67 and p67C induce similar levels of immunity to ECF, we embarked on the evaluation of different nanoparticle–antigen delivery platforms with p67C, as it is an easier antigen to work with than p67, and nanoparticle antigens are reported to be more immunogenic than soluble ones [11,23,24,25]. In order to compare data between cattle and antigen platforms, we developed a semi-quantitative method to measure anti-p67C antibody responses [23,26]. Presenting p67C in a multimerised and organised array played a crucial role in amplifying immune responses, and several innovative methods have been employed to achieve this enhancement.

The adsorption of p67C (70 μg/dose) onto silica vesicles, specifically SV-140-C_18_, resulted in an increase in antibody and cellular response specific to the antigen after three doses compared to the traditional delivery of protein in a soluble form (s-p67C) [24]. In the same study, another strategy involved genetic fusion of p67C (70 μg/dose) to the hepatitis B core antigen (HBcAg), generating virus-like particles. This platform achieved 2–3-fold higher antibody responses after three doses (p67C, 70 μg/dose; HBcAg-p67C total protein, 300 μg/dose) but lower T-cell responses compared to soluble p67C. The last study using p67C as a model antigen used a two-component self-assembled nanoparticle platform technology. Three doses of p67C-I53-50 were administered (p67C, 70 μg/dose; total protein, 424 μg/dose), generating high antibody and T-cell responses using only one platform technology [23]. In all cases, the immunogens were adjuvanted with ISA206VG (Seppic).

The multimerisation of p67C has been demonstrated to be very powerful in enhancing the specific immune responses to the antigen. However, all these technologies required the use of commercial adjuvants for their efficacy, and none of them offered a simple plug-and-play opportunity to include multiple antigens or immunomodulators in the nanoparticle to enhance the immune response. SNAP provides an exceptional platform specifically designed as a rapid and flexible system for testing vaccine candidates and immunomodulators, overcoming the challenges of traditional delivery systems [15].

Here, we present the evaluation of four different preparations of cobalt porphyrin–phospholipid (CoPoP) combined with immunomodulators to enhance the immune responses of the *Theileria parva* model antigen p67C in bovine and mouse models. The use of CoPoP only (p67C-CA) or combined with PHAD (p67C-CP) elicited a lower immune response than adjuvanted s-p67C in cattle. In contrast, the incorporation of saponin (QS-21) in the nanoparticles (either p67C-CQ or p67C-CPQ) elicited antibody responses approaching those of traditional adjuvanted s-p67C in cattle. However, these formulations failed to induce a p67C-specific cellular response. Notably, p67C-CPQ elicited a significantly higher antibody response in mice (2000-fold increase), highlighting the need to select the best immunomodulators and optimise their dose for effective delivery in large livestock animals.

Overall, the SNAP platform shows potential as a versatile and easy-to-formulate vaccine delivery system. However, optimisation of both the dose and the type of immunomodulatory adjuvants incorporated into the CoPoP particles will be critical for advancing this technology in large livestock, particularly for complex pathogens such as *Theileria parva*, the causative agent of East Coast fever (ECF).

## 2. Materials and Methods

### 2.1. Liposome Preparation

The liposomes were prepared by the ethanol injection method as previously described [17]. Liposomes were synthesised using porphyrin–phospholipid (PoP) conjugates chelated with cobalt (CoPoP). Immune modulators, a synthetic version of monophosphoryl lipid A (PHAD) and/or QS-21 (purified triterpene glycoside saponin from the *Quillaja saponaria* tree), were incorporated into CoPoP liposomes, resulting in the following formulations: CoPoP alone (CA), CoPoP with PHAD (CP), CoPoP with QS-21 (CQ), and a combination of CoPoP with PHAD and QS-21 (CPQ). The specific quantities for each formulation are detailed in Supplementary Table S2. Liposome sizes, zeta potential, and polydispersity index were determined by dynamic light scattering with a NanoBrook 90 Plus PALS instrument (Brookhaven Instrument Corporation, Holtsville, NY, USA) after 200-fold dilution in PBS.

### 2.2. Evaluation of Particle Formation with p67C

**Figure 1:**
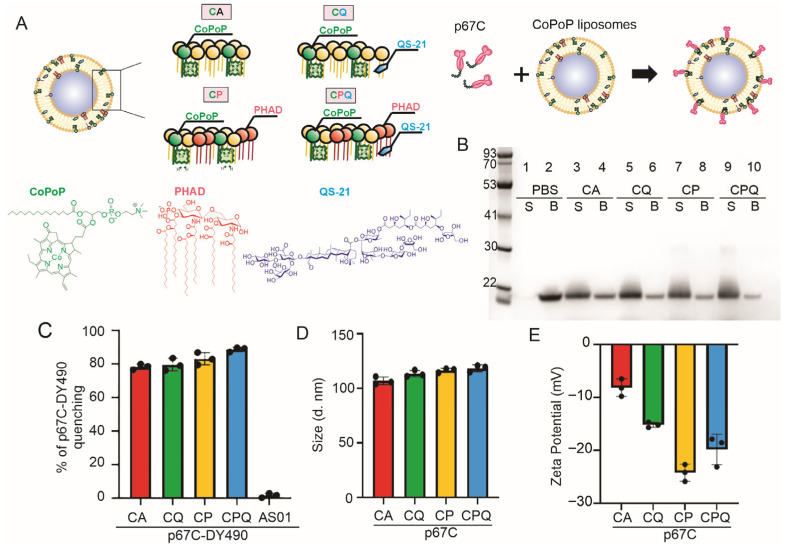
p67C-CoPoP liposome nanoparticle characterisation. (**A**) Schematic representation of the generation of CoPoP liposomes with p67C antigen, including the schematic illustrations of the chemical structures of the key adjuvanting molecules. (**B**) Binding capacity of the p67C antigen to CoPoP liposomes after 3 h incubation in the dark, analysed using HisPur™ Ni-NTA Magnetic Beads and assessed by SDS-PAGE. Formulations included: CA (CoPoP only), CQ (CoPoP/QS-21), CP (CoPoP/PHAD), and CPQ (CoPoP/PHAD/QS-21), compared to soluble p67C (PBS). Non-liposome-bound antigen is shown in lanes “B”, while liposome-bound is shown in lanes “S”. The uncropped blots are shown in Supplementary Materials Figure S1. (**C**) Binding capacity of fluorophore-labelled p67C-DY490 to CoPoP liposomes (CA, CQ, CP, and CPQ) was measured and compared with p67C bound to AS01, and (**D**) size and (**E**) zeta potential of p67C bound to CoPoP liposomes were measured by DLS.

The particleisation of CoPoP liposomes with p67C antigen was enabled by mixing His-tagged fusion p67C antigen (80 µg/mL, equivalent to 56 µg/mL of p67C) at a volume ratio of 1:1 (*v*/*v*) with CoPoP liposomes (320 µg/mL of CoPoP), resulting in an antigen-CoPoP mass ratio of 1:4. The mixture was incubated for 3 h at room temperature in the dark, as previously described [17] (Figure 1A and Supplementary Materials Figure S1 and Table S1). The His-tagged p67C antigen was sourced from GenScript Biotech (Piscataway, NJ, USA), free of endotoxins and with an estimated purity of 95%. The protein binding strength of His-tagged p67C to four different CoPoP liposomes was evaluated using HisPur™ Ni-NTA Magnetic Beads (Thermo Fisher Scientific, Waltham, MA, USA) as previously described [17]. Briefly, 5 μL of HisPur™ Ni-NTA Magnetic Beads were incubated with 25 μL of the different nanoliposome mixtures containing 40 μg/mL of antigen in p67C-CoPoP liposomes or with 25 μL of p67C antigen in PBS as a control. After incubation for 30 min at RT, the mix was gently shaken for 10 min and placed on a magnetic separator to isolate unbound free antigen. The fraction of p67C protein not forming nanoparticles in the mix is expected to bind to the magnetic beads. The samples were then analysed in Novex 4–12% Bis-Tris acrylamide gel (Invitrogen, Carlsbad, CA, USA), stained with ProtoBlue Safe staining (National Diagnostics, Atlanta, GA, USA), and visualised using the GelDoc Go Gel Imaging System (Bio-Rad, Hercules, CA, USA).

### 2.3. Quenching Assay to Assess Binding Capacity of Liposomes to p67C

To measure the binding efficiency of the antigen to the liposomes, the His-tagged fusion p67C protein (1 mg/mL, 200 μL) was dialysed twice against 1 L of 50 mM sodium bicarbonate buffer (pH 9) at 4 °C overnight, using a Slide-A-Lyzer Dialysis Cassette G2 (Thermo Scientific). Following dialysis, the antigen was labelled with 10 μL of 1 mg/mL of DY-490-NHS-Ester (Dyomics, Jena, Germany) for 1 h at RT with continuous stirring (p67C-DY490). To eliminate any unbound dye, the labelled antigen underwent three rounds of dialysis against 1 L of PBS at 4 °C. The p67C-DY490 was then diluted to a concentration of 80 μg/mL and incubated with 320 μg/mL of CoPoP (CA), CoPoP/PHAD (CP), CoPoP/QS-21 (CQ), CoPoP/PHAD/QS-21 (CPQ) or AS01 (from GlaxoSmithKline (GSK), Rixensart, Belgium), a commercially available liposomal adjuvant containing MPLA and QS-21, to measure fluorescence quenching after a three-hour incubation period. Samples were diluted 10-fold before measuring. Fluorescence quenching occurs when p67C-DY490 binds to the liposomes, which was measured using a Tecan Safire microplate fluorescence reader (Tecan Group Ltd., Männedorf, Switzerland) at an excitation wavelength of 490 nm and emission wavelength of 515 nm. The results are presented as the fluorescence intensity (FI) of the liposome-bound p67C-DY490 compared with the fluorescence of free antigen in PBS, using the formula [1-FIp67C-DY490_liposomes/FI free p67C-DY490 in PBS]X100.

### 2.4. Cattle Immunogenicity Experiment

Fourteen-month-old Friesian/Holstein (*Bos taurus*) male cattle were sourced from the International Livestock Research Institute (ILRI) Kapiti Research Station in Machakos County, Kenya. Animals were screened for prior exposure to *Theileria parva* and *Theileria mutans* using a serology assay, as previously described [27]. Twenty cattle were randomly assigned to four study groups, with five animals per group, balanced by their weight and age (Table 1). Animal groups were blinded to all personnel involved in the experiment by the ILRI Data and Research Methods Unit (DRMU). Liposomes containing p67C were prepared by mixing 0.4 mg/mL of His-tagged fusion p67C antigen (equivalent to 0.28 mg/mL of p67C sequence) with 1 mg/mL of CoPoP liposomes at a volume ratio of 1:2, and the mixtures were incubated for 3 h at room temperature (Figure 1A). Each animal received two intramuscular injections (neck region near the parotid lymph node) administered 28 days apart of 750 μL of p67C-CoPoP liposome, with a constant of 70 μg of actual p67C sequence, to keep consistency with previous experiments [23,24], and 500 μg of CoPoP liposomes in all formulations, with or without the presence of immunomodulators (Table 1 and Supplementary Table S2). Post-immunisation, the animals were closely monitored for adverse effects, with observations made every hour for the first eight hours and the following day. The study spanned 84 days, and animals were sampled as detailed in the manuscript. The cattle were housed at ILRI’s Nairobi campus farm facilities, and the experiment was approved by the Institutional Animal Care and Use Committee at ILRI (IACUC number 2022-19).

### 2.5. Murine Immunogenicity Experiment

Six-week-old female CD-1 mice (Envigo RMS LLC, Indianapolis, IN, USA) were intramuscularly injected with 50 μL of immunogen on days 0 and 21 containing 2 μg of p67C antigen combined with CoPoP/PHAD/QS-21 (CPQ) or Alum. Group A immunogen was prepared by mixing CPQ (320 μg/mL of CoPoP) with His-tag fusion p67C (100 μg/mL, equivalent to 70 µg/mL of p67C sequence) at a 1:4 mass ratio of antigen to CoPoP and incubated at room temperature for 3 h prior to injection. Group B immunogen was prepared by mixing p67C antigen with Alhydrogel^®^ 2% aluminium gel (InvivoGen, San Diego, CA, USA). The Alum suspension was diluted in HEPES buffer to a final concentration of 3 mg/mL and subsequently combined at a 1:1 volume ratio with the His-tag fusion p67C antigen (100 μg/mL, equivalent to 70 µg/mL of p67C sequence) solution. Blood samples were collected from the facial vein (submandibular region) using a lancet on days 0 and 42 post-immunisation. The mice were housed at the Comparative Medicine and Laboratory Animal Facilities (CM-LAF) of the University at Buffalo, State University of New York, under IACUC Protocol #BME05044Y.

### 2.6. Measurement of p67-Specific IgG and Specificity to 15-Mer Overlapping p67C Synthetic Peptides

ELISA was used to evaluate the presence of antibodies in both cattle and mouse serum samples. For the cattle experiment, the ELISA assay was conducted at the International Livestock Research Institute (ILRI) laboratories as previously described [23]. Briefly, 96-well Maxisorp plates (Nunc, Roskilde, Denmark) were coated with 0.5 μg/mL of recombinant p67C. Plates were then blocked, and sera were added to the plates in a 10-fold serial dilution starting from 1/1000 to 1/100,000, in duplicates, and incubated for 2 h at 37 °C. Mouse anti-bovine IgG:HRP (MCA2439P, Bio-Rad) was used for the detection of p67C-specific antibodies. The reaction was developed using tetramethylbenzidine (TMB Plus 2, Kem-en-Tec, Taastrup, Denmark) and stopped with 0.5 M sulfuric acid (Honeywell-Fluka, Charlotte, NC, USA). The absorbance was measured at 450 nm using a Synergy HT ELISA reader (BioTek, Winooski, VT, USA). p67C-specific antibodies in the serum samples were quantified by extrapolating the optical density signal to a standard curve generated from affinity-purified p67C-specific antibodies. Results are expressed in μg/mL after adjusting for the dilution factor.

p67C-specific antibody titres in mouse sera were measured by ELISA at the Comparative Medicine and Laboratory Animal Facilities (CM-LAF) of the University at Buffalo. Briefly, Flat-Bottom Immuno MaxiSorp Nonsterile 96-Well Plates (Nunc, Roskilde, Denmark) were coated with 1 μg/mL of p67C in Carb/Bicarb buffer (pH 9.6) for 2 h at 37 °C. After coating, the plates were blocked with 2% BSA in PBS containing 0.1% Tween-20 (PBS-T) for 2 h at 37 °C. Mouse sera, diluted (100-time dilution, following 10-time serial dilution till 10^5^ dilution) in PBS-T containing 1% BSA, were then added to the wells and incubated for 1 h at 37 °C. Anti-mouse IgG-HRP (Cell Signalling Technology, Danvers, MA, USA), diluted 1:2000 in 1% BSA-PBST, was used for the detection of p67C-specific mouse antibodies and incubated for 30 min at 37 °C. Plates were washed six times with PBS-T in between every step. The reaction was developed by adding the tetramethylbenzidine (TMB, VWR, Radnor, PA, USA) solution and stopped by adding 1 M HCl solution. The p67C-specific titres were defined as the reciprocal serum dilution at which the absorbance at 450 nm exceeded background by greater than 1.0 absorbance units.

Peptide epitope specificity of the immunised bovine sera was evaluated using ten overlapping biotinylated 15-mer peptides to p67C (Mimotopes Pty, Victoria, Australia [28]), as previously described [23]. Serum samples were diluted to 0.1 μg/mL of p67C-specific IgGs using blocking buffer and complemented with naïve bovine serum to adjust the volume of bovine serum present in the reaction. The results are presented as a heatmap of the percentage of the signal of a particular peptide compared to the sum of signals from all peptides in a sample.

### 2.7. PBMC, CD4^+^ and CD8^+^ T-Cell IFN-γ ELISpot

PBMCs were isolated using a Ficoll gradient, and CD4^+^ and CD8^+^ T-cells and CD14^+^-enriched fractions were obtained using magnetic beads (Milteny Biotec, Bergisch Gladbach, Germany) coated with anti-bovine CD4 or CD8 ascites (ILRI mouse hybridoma clones ILA11 and ILA51, respectively) or goat anti-human CD14 IgG-conjugated beads (Milteny Biotec). Both cell isolation and enrichment were performed as previously described [29].

Cellular responses were assessed by IFN-γ ELISpot using PBMCs at day 49 and CD4^+^ or CD8^+^ T-cells (complemented with 10% of CD14^+^ cells acting as antigen-presenting cells) at day 42 after immunisation, as previously described [29]. Three stimuli were used: 15-mer p67C peptides overlapping by seven amino acids, 25-mer p67C peptides overlapping by 16 amino acids (both peptide pools at 2 μM, Mimotopes Pty), and p67C recombinant protein at 2.5 μM (GeneScript Biotech, Piscataway, NJ, USA). Controls included media and irrelevant protein and peptides (p67N from Genescript and p67N 25-mer from Mimotopes Pty) as negative controls, with Con A at 2.5 μg/mL serving as a positive control. Cells at 2.5 × 10^5^ cells/well were tested in duplicate, incubating with stimuli for 20 h at 37 °C. Results are expressed as the fold change in IFN-γ-secreting cells using a specific stimulus compared to the non-stimulated cells (incubated with media).

### 2.8. In Vitro Stimulation of Bovine PBMCs with Quil-A

Heparinised blood for PBMC isolation was sampled in Alsever’s solution from three *Theileria parva* naïve bulls (*Bos taurus*), following approval from the Institute of Animal Care and Use Committee at ILRI (IACUC number 2022-23). The PBMCs were isolated using a Ficoll gradient, then resuspended in RPMI 1640 media (Merk, Darmstadt, Germany), supplemented with 10% heat-inactivated foetal calf serum (FCS; Gibco, Thermo Scientific, Waltham, MA, USA), 2 mM L-glutamine (Merk), 1 μg/mL gentamicin, 100 UI/mL penicillin, and 100 μg/mL streptomycin (all from Carl Roth, Karlsruhe, Germany). For each of the three PBMCs, 2 × 10^6^ cells per well were seeded in Costar 96-well round-bottom plates (Corning Inc,. Corning, NY, USA) and stimulated with increasing concentrations of Quil-A (kindly donated by Afrigen Biologics, Cape Town, South Africa): 0.5, 1, 3, 4, 5, and 6 µg/mL for 24 h at 37 °C. Complete RPMI was used as a negative control for stimulation. After incubation, supernatants were collected and stored at −80 °C for cytokine analysis. Cell viability after Quil-A stimulation was assessed using fluorescence-activated cell sorting (FACS). Briefly, 10^6^ PBMCs per well were stained with near-IR dead cell stain (Invitrogen) according to the manufacturer’s instructions. Heat-treated PBMCs (56 °C for 30 min) served as the positive control for dead cells, while unstimulated PBMCs incubated in complete RPMI media were used to establish the baseline of cell death of PBMCs in cell culture. Samples were measured on a BD Canto II (BD Biosciences, San Jose, CA, USA), and data were analysed using FlowJo software (version 10.8.1, BD Life Sciences, Ashland, OR, USA).

### 2.9. Cytokine and Chemokine Analysis of PBMC Supernatant Following Quil-A Stimulation

Thawed supernatants from PBMCs stimulated with Quil-A were analysed for cytokine and chemokine secretion using a bovine-customised 10-plex MILLIPLEX kit (MilliporeSigma, Burlington, MA, USA), including IFN-γ, IL-1α, IL-1β, IL-4, IL-6, IL-8, IL-10, IL-17A, IL-36RA, and TNF-α, following the manufacturer’s instructions. Briefly, lyophilised standards were reconstituted with 250 µL deionised water and prepared in a six-step, five-fold serial dilution. Supernatant samples were diluted 1:2 in assay buffer, and 25 µL of each sample, standard, and control was added to designated wells, followed by 25 µL of magnetic bead mix. Plates were sealed and incubated at 750 rpm for 2 h at room temperature. After three washes with 100 µL of wash buffer, 25 µL of detection antibodies were added to each well, and the plates were sealed and incubated for 1 h at 750 rpm at room temperature. Without further washing, 25 µL of Streptavidin-Phycoerythrin was added to each well and incubated for 30 min on a shaker at 750 rpm. Plates were then washed three times, and magnetic beads were resuspended in 100 µL of wash buffer. Samples were acquired on a Luminex 200^TM^ system (Luminex Corporation, Austin, TX, USA), with all plates passing quality control as per the manufacturer’s specifications. Data were obtained as median fluorescence intensities (MFI) recorded in the Luminex system and processed by Luminex xPONENT software version 4.3 (Luminex Corporation, Austin, TX, USA).

### 2.10. Statistical Analysis

Due to immunogenicity data in cattle and mice not fulfilling the assumptions of normality and homogeneity of variance, non-parametric methods were used. For comparisons of p67C-specific total IgG antibody responses on study day 42 across cattle groups, the Kruskal–Wallis test was used, followed by a pairwise comparison using Dunn’s multiple comparison test with Bonferroni correction. For mouse groups, comparisons were performed using the Mann–Whitney test. All statistical analyses were conducted using GraphPad Prism version 10.4.2 (GraphPad Software, San Diego, CA, USA), and a *p*-value < 0.05 was considered statistically significant.

## 3. Results

### 3.1. Recombinant His-Tagged p67C Spontaneously Binds to CoPoP Liposomes

The binding of recombinant His-tagged p67C to CoPoP liposomes was evaluated using the HisPur™ Ni-NTA Magnetic Bead assay. After a 3 h incubation, the unbound His-tagged p67C would be found in the elution fraction (*B*), while p67C bound to CoPoP liposomes would be found in the flowthrough (*S*) (Figure 1B). p67C successfully bound to all the CoPoP liposomes in an equivalent capacity, as evidenced by the presence of a significant portion of p67C in the flowthrough fraction (*S*) of p67C-CA, p67C-CQ, p67C-CP and p67C-CPQ (Figure 1B, lanes 3–10). In contrast, soluble His-tagged p67C remained unbound and was found in the magnetic bead elution fraction (*B*) (Figure 1B, lanes 1–2). The binding capacity of p67C to CoPoP/PHAD/QS-21 liposomes was further confirmed using a DY-490 fluorescence-quenching assay, where it was confirmed that around 80% of p67C was bound to the CoPoP liposomes (Figure 1C). CoPoP/PHAD/QS-21 (CPQ) presented a slightly better binding capacity but not significantly different from the other CoPoP liposomes. As expected, no p67C binding to AS01 adjuvant was detected.

Liposome size was evaluated, and homogeneity and zeta potential of His-tagged p67C-liposome mixtures were measured by dynamic light scattering (DLS). The particle size of p67C-CoPoP liposomes was around 100 nm (Figure 1D), as previously described using other antigens [30], and the zeta potential of the p67C-liposomes was slightly negatively charged (Figure 1E). The p67C-bound CoPoP liposomes maintained a polydispersity index (PDI) below 0.5, indicating homogeneity of particle size in the mix after binding.

### 3.2. Comparative Analysis of the p67C-Specific Antibody and Cellular Responses of Cattle Immunised with p67C-CoPoP Liposomes

**Figure 2:**
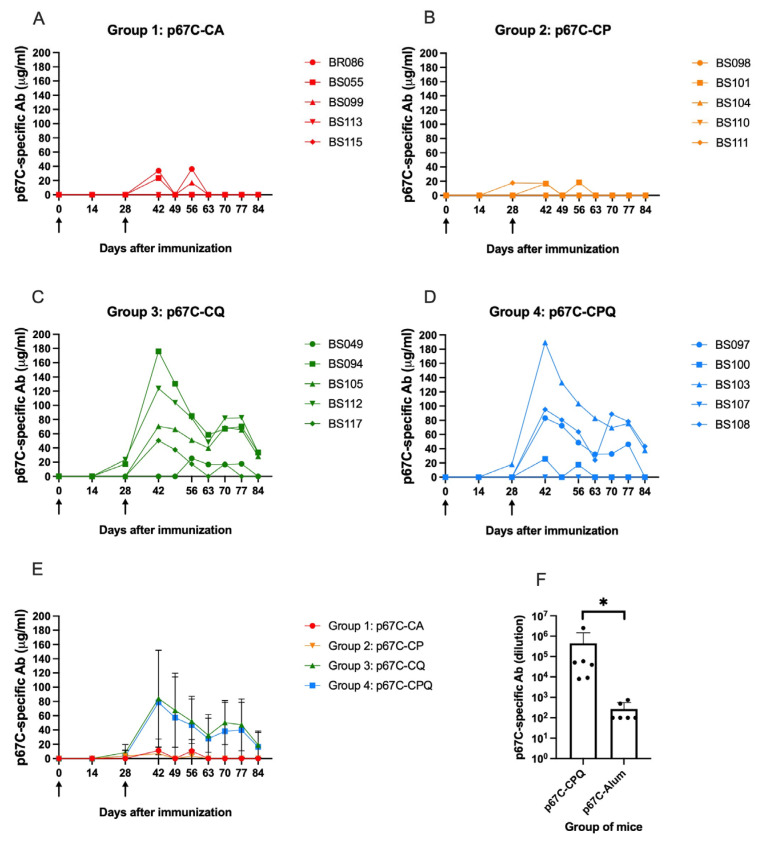
IgG responses in both cattle and mice immunised with p67C using different CoPoP liposomes. Serum samples from cattle immunised with (**A**) p67C-CA, (**B**) p67C-CP, (**C**) p67C-CQ and (**D**) p67C-CPQ were evaluated using a p67C quantitative ELISA. Kinetics of individual animals are presented in panels (**A**–**D**), and the mean and standard error of the groups are presented in panel (**E**). Antigen inoculation times are indicated with black arrows in the x-axis. (**F**) Semi-quantification of antibody titres at day 42 in mice immunised with p67C-CPQ or p67C-Alum is presented as the mean of the last positive dilution and standard error per group. Significance is represented by a “*” for *p* < 0.05.

Four groups of five cattle were immunised with the p67C antigen bound to different CoPoP liposomes (Table 1) to assess the capacity of SNAP technology to enhance the immunogenicity of p67C. Each animal received two intramuscular doses of 70 μg of p67C, administered 28 days apart. No adverse reactions were observed at the injection sites after either the first or second administration of the p67C-CoPoP liposome preparations. The ratio of seroconversion in the different groups was variable, with two and three animals seroconverting from the five vaccinated in Group 1 (p67C-CA) and Group 2 (p67C-CP), respectively (Figure 2A,B), and five and four animals seroconverting in Group 3 (p67C-CQ) and Group 4 (p67C-CPQ), respectively (Figure 2C,D).

In line with these findings, and despite the large variability between the outbred cattle in the same group, on average, Group 4 (p67C-CPQ) and Group 3 (p67C-CQ) cattle developed higher antibody titres (*p* < 0.05; day 42 titres: 78.8 ± 73.5 μg/mL and 84.2 ± 67.9 μg/mL, respectively; Figure 2C–E) than animals in Group 1 (p67C-CA at day 42 titres: 11.42 ± 16.1 μg/mL) or Group 2 (p67C-CP at day 42: 6.7 ± 9.2 μg/mL) (Figure 2A,B,E). The presence of QS-21 (saponin) in the CoPoP liposomes seems to be essential for the efficacious generation of p67C-specific antibodies in cattle. However, the antibody titres developed by animals in Groups 3 and 4 were still on average two times lower than the titres in animals vaccinated with soluble p67C adjuvanted with Montanide ISA206VG at the same time in the experiment, as previously reported [23].

**Figure 3:**
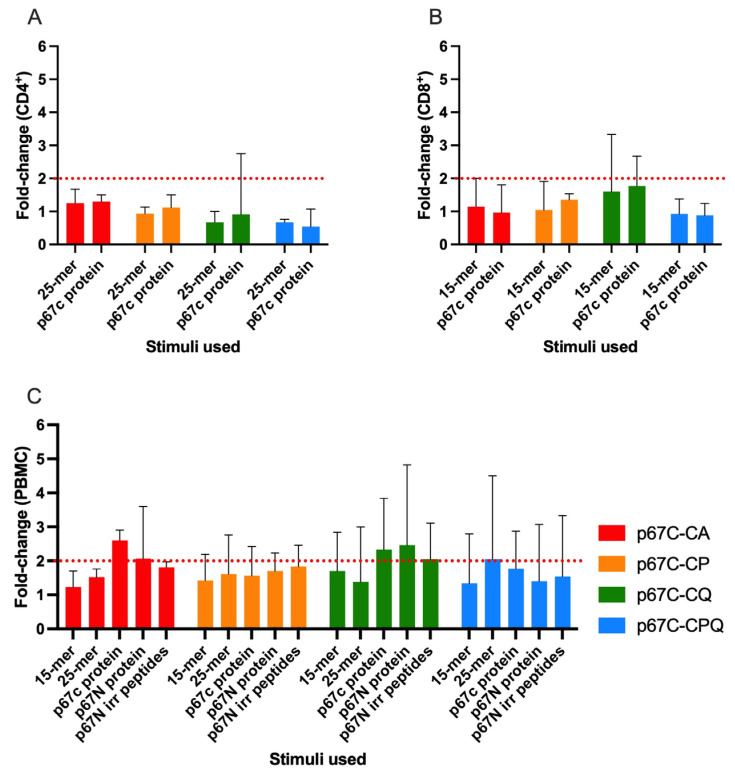
Cellular response specific to p67C by means of the INFγ-ELISpot assay. (**A**) CD4^+^ T-cell, (**B**) CD8^+^ T-cell and (**C**) PBMC responses to 15-mer and 25-mer based on the p67C sequence and p67C protein used as stimuli. Irrelevant peptides and proteins based on the sequence of p67N (N-terminal portion of p67) were used as negative controls. Cells were stimulated for 20 h at 37 °C. Results are presented as the group mean ± standard deviation (SD) of the fold change in the number of spot-forming units compared to the cells stimulated with media only (background). Dotted red lines indicate the cut-off from which the response can be considered different from the background (fold change of 2).

The cellular response specific to p67C was evaluated by means of an IFN-γ ELISpot assay using MACS-isolated CD8^+^ T-cells complemented with CD14^+^ cells serving as APCs and CD4^+^ T-cells, collected at day 42 (two weeks after the antigen boost). The response to any of the stimuli (15-mer, 25-mer peptide pools or p67C protein) did not differ from the background response (cells stimulated with media only, Figure 3A,B) in any of the T-cell types. To elucidate if the undetectable cellular response was due to a lack of cellular interaction, the IFN-γ ELISPOT was repeated at day 49 using PBMCs (Figure 3C). Again, the responses to the stimuli did not differ from the background (cells stimulated with irrelevant protein or peptides based on the sequence of p67N), concluding that none of the p67C-CoPoP liposome formulations were able to trigger a detectable cellular response in cattle.

### 3.3. p67C-CoPoP/PHAD/QS-21 (p67C-CPQ) Induces High IgG Antibody Titres in Mice

In the interest of ascertaining the appropriateness of the CoPoP technology for the delivery of p67C, a poorly immunogenic antigen, a group of six mice was immunised with p67C-CoPoP/PHAD/QS-21 (p67C-CPQ) at a mass ratio of p67C antigen to CoPoP of 1:4, which was similar to the antigen-to-CoPoP mass ratio in previous successful experiments [20]. A second group of mice received p67C adjuvanted with Alum (p67C-Alum). None of the mice had adverse reactions to the injected material. Sera were collected on days 0 and 42 to semi-quantify the titre of p67C-specific antibodies. Mice that received p67C combined with CoPoP/PHAD/QS-21 (p67C-CPQ) successfully developed significantly higher antibody titres compared to the mice administered with p67C-Alum (around 2000 times higher, *p* < 0.05; Figure 2F). These findings highlight the enhanced immunogenicity of p67C when formulated with CoPoP liposomes combined with immunomodulators in mice and emphasise the critical importance of selecting appropriate liposome components and optimising their concentrations for each target animal species.

### 3.4. Anti-p67C Antibody Specificity to Linear Synthetic Peptides in Cattle Sera

**Figure 4:**
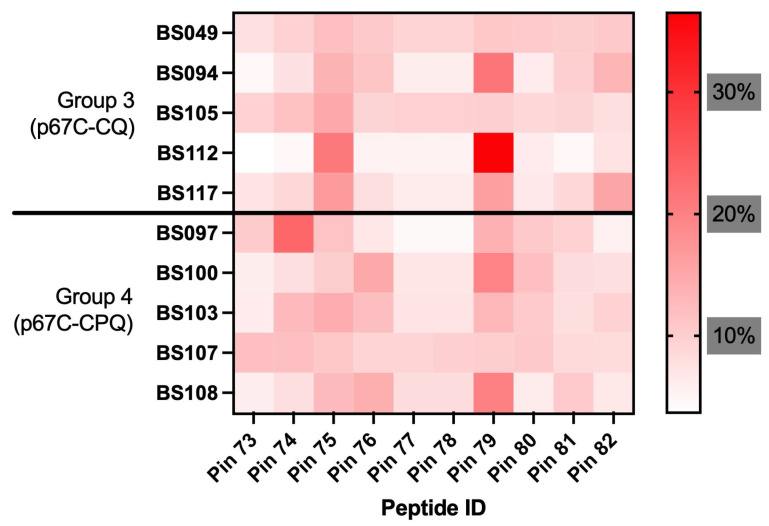
IgG reactivity to p67C 15-mer overlapping peptides. Day 42 sera from cattle immunised with p67C-CoPoP liposomes: Group 3 (p67C-CQ) and Group 4 (p67C-CPQ) were analysed by means of an ELISA assay. Results are presented as a heat map of the relative reactivity compared to the sum of responses against all peptides for each animal.

To gain deeper insights into the immune responses elicited, sera collected on day 42 (corresponding to the peak of antibody response) from cattle immunised with p67C-CQ (Group 3) or p67C-CPQ (Group 4) were analysed for reactivity against a panel of ten 15-mer peptides overlapping by seven amino acids [28], covering the p67C sequence (Figure 4). In line with previously published experiments using soluble p67C [24], the majority of the response (Group 3, 19.0%, and Group 4, 15.2% of the overall average signal) was directed to the linear sequence of pin 79 (SERQPSLGPSLVITD), coinciding with the sequence recognised by a monoclonal antibody, mAR21.4, with neutralising capacity [28,31]. Interestingly, Group 3 also exhibited a substantial proportion of the overall antibody signal directed against pin 75 (EEEVKKILDEIVKDP), accounting for an average of 15.7% of the total response (Figure 4). This epitope is also recognised by a monoclonal antibody m38.9 that lacks neutralising capacity [28]. In a separate study, cattle vaccinated with p67C-I53-50 self-assembled nanoparticles, which conferred protection against *Theileria parva* challenge and induced neutralising antibodies, also showed a strong response targeting pin 75 [23].

### 3.5. In Vitro Cytokine Response Induced by Quil-A (Saponin)

**Figure 5:**
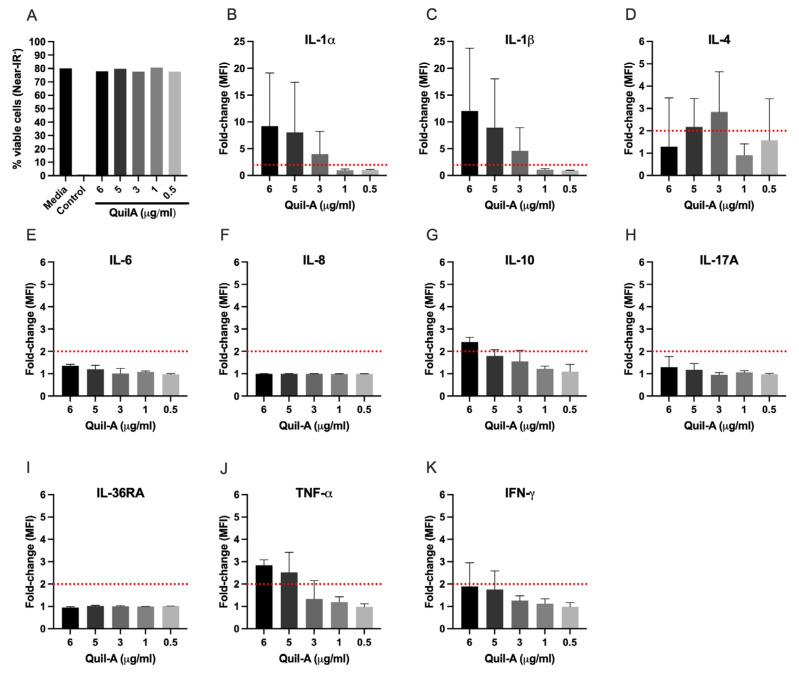
Cell viability and cytokine/chemokine expression profile in bovine PBMCs stimulated with Quil-A (saponin). (**A**) Cell viability was measured by staining with near-IR fixable dye and measured by FACS analysis on PBMCs stimulated with different concentrations of Quil-A for 24 h. Non-treated cells and cells incubated for 30 min at 56 °C were used as negative and positive controls for near-IR staining, respectively. Results are presented as the average of the viability of PBMC from 3 donor cattle. (**B**–**K**) Cytokine/chemokine expression profile in bovine PBMCs treated with Quil-A (saponin) at increasing concentrations for 24 h. The net median fluorescence intensity (MFI) values were measured, and the results are presented as the mean and standard deviation (SD) of the fold-changes relative to non-stimulated cells (PBMCs cultured in media only). A red dashed line represents the cut-off threshold for differential cytokine expression compared to non-treated cells.

To characterise the type of signalling cascade triggered by saponins in cattle, PBMCs from three naïve donors were stimulated with increasing concentrations of the saponin, Quil-A (0.5, 1, 3, 5, and 6 µg/mL), for 24 h at 37 °C. Cell viability and cytokine response were evaluated. The cell treatment with increasing concentrations of Quil-A did not affect cell viability (Figure 5A). The signalling pathway triggered by Quil-A had a remarkable pro-inflammatory signature. The expression of IL-1α and IL-1β (Figure 5B and Figure 5C, respectively) progressively increased when stimulated with increasing concentrations of Quil-A (9-fold and 12-fold increase at 6 μg/mL, respectively). A more moderate increase in expression was also observed in TNFα (2.9-fold, Figure 5J), another pro-inflammatory cytokine, and IL-10 (2.4-fold, Figure 5G), with anti-inflammatory activity. The other cytokines/chemokines measured (IL-4, IL-6, IL-8, IL-17A, IL-36RA and IFN-γ) did not present substantial differences in expression compared to non-stimulated bovine PBMCs (Figure 5D, Figure 5E, Figure 5F, Figure 5H, Figure 5I and Figure 5K, respectively).

## 4. Discussion

Commercially available livestock vaccines predominantly rely on the use of inactivated or live attenuated pathogens. These more traditional technologies pose several limitations, such as insufficient thermostability, the potential for animals to act as vectors, and complex administration procedures. In recent years, the field of vaccinology has rapidly evolved, expanding the offer of delivery systems for humans, companion animals, and livestock [1,6]. The combination of different expertise in the context of “systems vaccinology” helped to enhance our understanding of the compatibility and performance of new technologies and compounds, paving the way for innovative vaccine strategies tailored to specific pathogens and host species. A prominent example of innovation is the SNAP technology, which represents a convergence of molecular biology, synthetic chemistry and lipid nanotechnology. Traditionally used for cancer immunotherapy, this platform has now been adapted as a versatile delivery system for subunit vaccines, enabling the conjugation of antigens and immunomodulators to the nanoliposome to enhance the immunogenicity of subunit vaccine candidates [15].

Here, we evaluated the performance of CoPoP (cobalt porphyrin–phospholipid) liposomes combined with immunomodulators (QS-21 and PHAD) to enhance the immunogenicity of p67C, the C-terminal part of the major surface antigen of *Theileria parva* sporozoites, p67 [22]. SNAP offers an easy plug-and-play option for vaccine candidate and immunomodulator screening; however, little has been done to assess its potential on large livestock animals. In this sense, p67C, with limited immunogenic capacity, is an ideal model antigen to test new technologies for vaccine delivery in cattle, since it allows the measurement of subtle variations in the immune response.

In cattle, and in comparison with our historical data, CoPoP liposomes did not perform better than soluble p67C (s-p67C) or previously evaluated nanoparticle technologies administered with a commercial emulsion adjuvant (Montanide ISA206 VG, Seppic) [23,24]. Antibody responses were a minimum of 2-fold lower when using the CoPoP liposome formulation that included QS-21 saponin (p67C-CQ and p67C-CPQ), and no detectable cellular response was observed with any of the evaluated CoPoP liposomes. However, the delivery of p67C-CPQ in mice generated 2000 times higher antibody titres than the delivery of the same antigen adjuvanted with Alum, highlighting the great potential of the technology in enhancing p67C immunogenicity.

The significant disparity in antibody titres, despite a 62.5-fold increase in the dose of adjuvanting molecules and a 35-fold increase in antigen dose in cattle, is likely to be multifactorial. First, it is important to consider the large disproportion in the immunomodulator-to-body-volume ratio. The dose of immunomodulators used in mice can easily reach immune saturation, while in cattle, the dose used is diluted in a larger volume (approximately 128 μg/kg in mice and 0.8 μg/kg in cattle of PHAD or QS-21) [32,33]. In line with this, the nanoparticles were injected intramuscularly in both species, and while the distance to the nearest draining lymph node is negligible in mice, it is considerable in cattle. The nanoparticles can become trapped in the dense muscle tissue, exacerbating the dilution factor of both the immunomodulators and the antigen [34,35]. These findings underscore the capital importance of optimising the dose of antigen and immunomodulators for use in large livestock animals.

At the molecular level, the suboptimal performance of p67C-CP (PHAD-only formulation) in cattle might indicate species-specific limitations in the TLR4 ligand recognition and signalling pathway. Structural divergence in the TLR4/MD-2 (myeloid differentiation protein 2) complex across species can significantly alter ligand-binding affinity. Specifically, sequence variations in the MD-2 may diminish its capacity to recognise PHAD in cattle compared to mice, hence decreasing the intensity of the signal [36,37,38]. Furthermore, while the TLR4/MD-2/CD14 complex is essential for optimal activation in the bovine model [37,39], murine studies suggest that CD14 might be dispensable for efficient signalling in certain contexts, highlighting a less restrictive TLR4 activation in mice than in cattle [40]. Importantly, both species might differ in the downstream pathway activation. Although both MyD88-dependent and -independent cascades are present in both species, the MyD88-dependent cascade appears to play a more dominant role in bovine immunity [36,38,41], and the activation of a specific pathway is ligand-specific [42]. Collectively, these differences highlight the need for further investigation to identify the most effective TLR4 agonist for maximising immunogenicity in cattle.

On the contrary, QS-21 might have a more universal adjuvant capacity by directly stimulating the NLRP3 inflammasome upon the loss of cellular homeostasis, without the need for receptor-ligand interaction [43,44,45]. Thus, QS-21 might be able to generate a broader and more potent signal, which is translated into higher antibody titres than PHAD. However, the formulation is lacking the synergistic effect that QS-21 and PHAD often have when administered as an immunostimulatory combo, as it happens after the administration of antigens combined with the AS01 adjuvant [43,46].

Although the saponin mode of action has been extensively studied in mice and human models, the cellular targets and signalling pathways triggered by saponins in cattle remain poorly defined. Our results suggest that a similar signal cascade might be stimulated. Bovine PBMCs stimulated in vitro for 24 h with increasing concentrations of Quil-A exhibited a transcriptional profile indicative of NLRP3 inflammasome activation [43,44,46]. Key signature pro-inflammatory cytokines, including *IL-1α*, *IL-1β*, and *TNF-α*, were markedly upregulated. Notably, the anti-inflammatory cytokine *IL-10* was also slightly induced, likely as a regulatory mechanism to counterbalance the heightened inflammatory response. Together, these results indicate that saponins can activate conserved innate immune pathways across species, overcoming the differences in the genetic background in a way that TLR4 agonist, PHAD, could not.

The use of saponins for full-length p67 and p67C immunisation in cattle was previously reported with very successful antibody responses (reviewed in [22]), demonstrating the efficacy of saponins in helping to generate an immune response to p67C in cattle. However, in those experiments, the type and dose of saponin used are unclear, making it difficult to make a fair comparison with the data presented here. In line with those results, saponins have been extensively used as adjuvants for human and livestock vaccines. Several commercial vaccines for humans used QS-21 as part of the adjuvant formulation. Some examples are Shingrix against herpes zoster [47] and Mosquirix against malaria [48] (both produced by GlaxoSmithKline (GSK)) and Nuvaxovid (produced by Novavax) against COVID-19 [49]. In livestock, the tendency is to use less refined saponins, such as Quil-A, to make the vaccines more cost-effective on several of the already registered vaccines, such as Zulvac BTV against Bluetongue [50] and Zulvac SBV against Schmallenberg [51] (both produced by Zoetis), as well as the trivalent/hexavalent FMD vaccine developed by the National Veterinary Institute of Ethiopia and FOTIVAX, produced by the Kenya Veterinary Vaccines Production Institute (KEVAVAPI) [52,53]—both against foot-and-mouth disease. Importantly, QS-21 was successfully used as the only immunomodulator in CoPoP liposomes for the delivery of SARS-CoV-2 RBD antigen, with increased antigen-specific polyfunctional T-cell responses compared with CoPoP alone [54].

From a qualitative perspective of the immune response induced by the liposomes containing QS-21 saponin, we observed that the antibody response generated to p67C in animals from Groups 3 (p67C-CQ) and 4 (p67C-CPQ) was similar to that generated by using soluble p67C (s-p67C). The predominant response was directed to a linear peptide recognised by the neutralising monoclonal antibody, mAR21.4 (pin 79, SERQPSLGPSLVITD) [28,31]. Interestingly, Group 3 exhibited a substantial portion of the antibody response directed towards a sequence also recognised by sera from cattle immunised with the protective p67C-I53-50, which has neutralising capacity (pin 75, EEEVKKILDEIVKDP) [23]. Therefore, we can conclude that the p67C antigen is efficiently presented when formulated into CoPoP liposomes. Even though p67C remains a promising antigen, it is important to note that other regions within p67 also exhibit protective potential (reviewed in [22]). Moreover, several additional antigens with neutralising capacity have been identified [55], supporting a broader antigenic focus rather than restricting research to a single target. In this context, it would be valuable to evaluate the performance of the SNAP technology for the delivery of multivalent vaccines against East Coast fever (ECF).

Taking all the results together, the data strongly suggest that the dosage of antigen and/or immunomodulators used in CoPoP liposomes needs further optimisation for efficacy in cattle. Future systematic studies should begin with in vitro evaluations, followed by carefully designed in vivo immunogenicity experiments, to explore the potential of alternative adjuvant molecules and doses. Emphasis should be placed on identifying formulations that are not only effective in enhancing the immune responses but also economically viable and scalable for use in livestock species. For example, active research in several cattle diseases is focusing on the use of new saponin compounds and formulations to increase potency and stability and reduce toxicity (reviewed in [43,56]), which could potentially be incorporated into CoPoP liposomes. Such efforts would help ensure that promising vaccine platforms, like SNAP, can be translated into practical tools for livestock vaccinology, where cost, accessibility and ease of deployment are critical factors for successful implementation [57,58]. The versatility of SNAP technology, functioning as a plug-and-play system for His-tagged proteins, combined with the lack of reactivity against the liposomal carrier, makes it a very suitable technology for vaccines that require repeated administration. In addition, CoPoP manufacturing has been demonstrated at the 100 g scale, highlighting its scalability and potential for cost-effective production. Overall, this technology represents an attractive investment for delivering subunit vaccines for high-value livestock such as cattle, including for neglected diseases like ECF.

## 5. Conclusions

This study represents the first evaluation of SNAP-based CoPoP liposome technology for subunit vaccine delivery in cattle. While the platform demonstrated remarkable immunogenicity in mice, its performance in cattle was unexpectedly limited, highlighting the challenge of translating results from small to large animals and the need to target the vaccine formulation to the species of interest.

Our results showed that none of the CoPoP formulations tested in this study could enhance the immunogenicity of p67C antigen beyond that achieved in previous studies where p67C was administered using established adjuvants. The marked discrepancy observed between mice and cattle underscores the critical influence of the antigen dose, the tissue architecture, and the species-specific innate immune response on vaccine efficacy. Mechanistically, the data suggest that QS-21 has a broader capacity to stimulate the immune system of different species, most probably by activating conserved innate immune pathways, likely through the inflammasome-associated mechanism. On the other hand, PHAD-mediated activation might be less effective in cattle. Importantly, the qualitative antibody response indicates that the presentation of the antigen in the liposomes remains intact, supporting the continuity of research on this platform.

Taken together, these results highlight the need for systematic studies evaluating alternative adjuvant molecules and doses in vitro and in vivo, with special emphasis on compounds that are economically viable and scalable for use in livestock species in LMICs. The inherent versatility and scalability of SNAP technology make it a promising candidate technology for further development, particularly for multivalent vaccine strategies targeting complex diseases such as East Coast fever.

## Figures and Tables

**Table 1:** Summary of immunogenicity animal groups in cattle and mouse experiments.

Animal Groups	Immunogen	Number of Doses	p67C/Dose (μg)	CoPoP/Dose (μg)	Adjuvant/Dose (μg)	Number of Animals
Cattle studies						
Group 1	p67C-CA	2	70	500	-	5
Group 2	p67C-CP	2	70	500	200 (PHAD)	5
Group 3	p67C-CQ	2	70	500	200 (QS-21)	5
Group 4	P67C-CPQ	2	70	500	200 (each PHAD and QS-21)	5
Mouse studies						
Group A	p67C-CPQ	2	2	8	3.2 (each PHAD and QS-21)	6
Group B	p67C-Alum	2	2	-	5.5 (Alum)	6

## Data Availability

The raw data supporting the conclusion of this article will be made available by the corresponding author on request.

## Supplementary Materials

The following supporting information can be downloaded at https://www.mdpi.com/article/10.3390/vaccines14050459/s1, Figure S1: Unmodified Figure 1B. SDS-PAGE showing the binding capacity of p67C antigen to CoPoP liposomes after 3 hours of incubation in the dark, compared to soluble p67C (PBS) and assessed by SDS-PAGE; Table S1: SDS-PAGE band intensities of p67C after binding assay: CA (CoPoP only), CQ (CoPoP-QS21), CP (CoPoP-PHAD), and CPQ (CoPoP-PHAD-QS21); Table S2: Components of CoPoP liposomes formulations used in the cattle experiment.

## References

[1] Fanelli A., Mantegazza L., Hendrickx S., Capua I. (2022). Thermostable Vaccines in Veterinary Medicine: State of the Art and Opportunities to Be Seized. Vaccines.

[2] Kristensen D.D., Lorenson T., Bartholomew K., Villadiego S. (2016). Can Thermostable Vaccines Help Address Cold-Chain Challenges? Results from Stakeholder Interviews in Six Low- and Middle-Income Countries. Vaccine.

[3] Perry B., Sones K. (2007). Poverty Reduction Through Animal Health. Science.

[4] Hagan T., Nakaya H.I., Subramaniam S., Pulendran B. (2015). Systems Vaccinology: Enabling Rational Vaccine Design with Systems Biological Approaches. Vaccine.

[5] Pulendran B., Li S., Nakaya H.I. (2010). Systems Vaccinology. Immunity.

[6] Allam A.M., Elbayoumy M.K., Ghazy A.A. (2023). Perspective Vaccines for Emerging Viral Diseases in Farm Animals. Clin. Exp. Vaccine Res..

[7] Dungu B., Donadeu M. (2025). Veterinary Vaccines and Vaccination: From Science to Action—Reflections for Change.

[8] STAR-IDAZ Global Veterinary Vaccinology Research and Innovation Landscape Survey Report, 2022. https://www.star-idaz.net/report/global-veterinary-vaccinology-research-and-innovation-landscape-survey-report/.

[9] Thomas S., Abraham A., Rodríguez-Mallon A., Unajak S., Bannantine J.P., Thomas S. (2022). Challenges in Veterinary Vaccine Development. Vaccine Design: Methods and Protocols, Volume 2. Vaccines for Veterinary Diseases.

[10] Russell F.A., Hutmacher D.W., Dargaville T.R., Beagley K.W. (2024). Vaccine Delivery: Overcoming the Challenges of Vaccinating Livestock and Wildlife. Vet. Vaccine.

[11] Bezbaruah R., Chavda V.P., Nongrang L., Alom S., Deka K., Kalita T., Ali F., Bhattacharjee B., Vora L. (2022). Nanoparticle-Based Delivery Systems for Vaccines. Vaccines.

[12] Gregory A.E., Titball R., Williamson D. (2013). Vaccine Delivery Using Nanoparticles. Front. Cell. Infect. Microbiol..

[13] Krishnamachari Y., Salem A.K. (2009). Innovative Strategies for Co-Delivering Antigens and CpG Oligonucleotides. Adv. Drug Deliv. Rev..

[14] Sadr S., Poorjafari Jafroodi P., Haratizadeh M.J., Ghasemi Z., Borji H., Hajjafari A. (2023). Current Status of Nano-vaccinology in Veterinary Medicine Science. Vet. Med. Sci..

[15] Zhou S., Luo Y., Lovell J.F. (2023). Vaccine Approaches for Antigen Capture by Liposomes. Expert Rev. Vaccines.

[16] Curley S.M., Putnam D. (2022). Biological Nanoparticles in Vaccine Development. Front. Bioeng. Biotechnol..

[17] Huang W.-C., Deng B., Lin C., Carter K.A., Geng J., Razi A., He X., Chitgupi U., Federizon J., Sun B. (2018). A Malaria Vaccine Adjuvant Based on Recombinant Antigen Binding to Liposomes. Nat. Nanotechnol..

[18] Mabrouk M.T., Huang W.-C., Deng B., Li-Purcell N., Seffouh A., Ortega J., Ekin Atilla-Gokcumen G., Long C.A., Miura K., Lovell J.F. (2020). Lyophilized, Antigen-Bound Liposomes with Reduced MPLA and Enhanced Thermostability. Int. J. Pharm..

[19] Song Y., Huang W.-C., Ivanochko D., Long C., Li Q., Zhou L., Julien J.-P., Miura K., Lovell J.F. (2025). 50-Fold Adjuvant and 20-Fold Antigen Vaccine Dose Sparing from Nanoliposome Display of a Stabilized Malarial Protein Antigen. ACS Nano.

[20] Sia Z.R., Roy J., Huang W.-C., Song Y., Zhou S., Luo Y., Li Q., Arpin D., Kutscher H.L., Ortega J. (2024). Adjuvanted Nanoliposomes Displaying Six Hemagglutinins and Neuraminidases as an Influenza Virus Vaccine. Cell Rep. Med..

[21] Lovell J.F., Baik Y.O., Choi S.K., Lee C., Lee J.-Y., Miura K., Huang W.-C., Park Y.-S., Woo S.-J., Seo S.H. (2022). Interim Analysis from a Phase 2 Randomized Trial of EuCorVac-19: A Recombinant Protein SARS-CoV-2 RBD Nanoliposome Vaccine. BMC Med..

[22] Nene V., Kiara H., Lacasta A., Pelle R., Svitek N., Steinaa L. (2016). The Biology of *Theileria Parva* and Control of East Coast Fever—Current Status and Future Trends. Ticks Tick Borne Dis..

[23] Lacasta A., Kim H.C., Kepl E., Gachogo R., Chege N., Ojuok R., Muriuki C., Mwalimu S., Touboul G., Stiber A. (2023). Design and Immunological Evaluation of Two-Component Protein Nanoparticle Vaccines for East Coast Fever. Front. Immunol..

[24] Lacasta A., Mody K.T., De Goeyse I., Yu C., Zhang J., Nyagwange J., Mwalimu S., Awino E., Saya R., Njoroge T. (2021). Synergistic Effect of Two Nanotechnologies Enhances the Protective Capacity of the *Theileria Parva* Sporozoite p67C Antigen in Cattle. J. Immunol..

[25] Zhao L., Seth A., Wibowo N., Zhao C.-X., Mitter N., Yu C., Middelberg A.P.J. (2014). Nanoparticle Vaccines. Vaccine.

[26] Hjerrild K.A., Jin J., Wright K.E., Brown R.E., Marshall J.M., Labbé G.M., Silk S.E., Cherry C.J., Clemmensen S.B., Jørgensen T. (2016). Production of Full-Length Soluble Plasmodium Falciparum RH5 Protein Vaccine Using a Drosophila Melanogaster Schneider 2 Stable Cell Line System. Sci. Rep..

[27] Katende J., Morzaria S., Toye P., Skilton R., Nene V., Nkonge C., Musoke A. (1998). An Enzyme-Linked Immunosorbent Assay for Detection of *Theileria Parva* Antibodies in Cattle Using a Recombinant Polymorphic Immunodominant Molecule. Parasitol. Res..

[28] Nene V., Gobright E., Bishop R., Morzaria S., Musoke A. (1999). Linear Peptide Specificity of Bovine Antibody Responses to P67 of *Theileria Parva* and Sequence Diversity of Sporozoite-Neutralizing Epitopes: Implications for a Vaccine. Infect. Immun..

[29] Steinaa L., Saya R., Awino E., Toye P. (2012). Cytotoxic T Lymphocytes from Cattle Immunized against *Theileria Parva* Exhibit Pronounced Cross-Reactivity among Different Strain-Specific Epitopes of the Tp1 Antigen. Vet. Immunol. Immunopathol..

[30] Huang W.-C., Deng B., Seffouh A., Ortega J., Long C.A., Suresh R.V., He X., Miura K., Lee S.-M., Wu Y. (2020). Antibody Response of a Particle-Inducing, Liposome Vaccine Adjuvant Admixed with a Pfs230 Fragment. npj Vaccines.

[31] Miersch S., Singer A.U., Chen C., Fellouse F., Gopalsamy A., Costa L.S.E., Lacasta A., Chege H., Chege N., Nene V. (2025). Molecular Characterization of a Synthetic Neutralizing Antibody Targeting P67 of *Theileria Parva*. Protein Sci..

[32] Nair A., Morsy M.A., Jacob S. (2018). Dose Translation between Laboratory Animals and Human in Preclinical and Clinical Phases of Drug Development. Drug Dev. Res..

[33] Nair A.B., Jacob S. (2016). A Simple Practice Guide for Dose Conversion between Animals and Human. J. Basic Clin. Pharm..

[34] Mueller S.N., Tian S., DeSimone J.M. (2015). Rapid and Persistent Delivery of Antigen by Lymph Node Targeting PRINT Nanoparticle Vaccine Carrier To Promote Humoral Immunity. Mol. Pharm..

[35] Martin J.T., Hartwell B.L., Kumarapperuma S.C., Melo M.B., Carnathan D.G., Cossette B.J., Adams J., Gong S., Zhang W., Tokatlian T. (2021). Combined PET and Whole-Tissue Imaging of Lymphatic-Targeting Vaccines in Non-Human Primates. Biomaterials.

[36] Vaure C., Liu Y. (2014). A Comparative Review of Toll-Like Receptor 4 Expression and Functionality in Different Animal Species. Front. Immunol..

[37] Lizundia R., Sauter K.-S., Taylor G., Werling D. (2008). Host Species-Specific Usage of the TLR4-LPS Receptor Complex. Innate Immun..

[38] Werling D., Jungi T.W. (2003). TOLL-like Receptors Linking Innate and Adaptive Immune Response. Vet. Immunol. Immunopathol..

[39] Sauter K.-S., Brcic M., Franchini M., Jungi T.W. (2007). Stable Transduction of Bovine TLR4 and Bovine MD-2 into LPS-Nonresponsive Cells and Soluble CD14 Promote the Ability to Respond to LPS. Vet. Immunol. Immunopathol..

[40] Su L., Athamna M., Wang Y., Wang J., Freudenberg M., Yue T., Wang J., Moresco E.M.Y., He H., Zor T. (2021). Sulfatides Are Endogenous Ligands for the TLR4–MD-2 Complex. Proc. Natl. Acad. Sci. USA.

[41] Cronin J.G., Turner M.L., Goetze L., Bryant C.E., Sheldon I.M. (2012). Toll-Like Receptor 4 and MYD88-Dependent Signaling Mechanisms of the Innate Immune System Are Essential for the Response to Lipopolysaccharide by Epithelial and Stromal Cells of the Bovine Endometrium1. Biol. Reprod..

[42] Zughaier S.M., Zimmer S.M., Datta A., Carlson R.W., Stephens D.S. (2005). Differential Induction of the Toll-Like Receptor 4-MyD88-Dependent and -Independent Signaling Pathways by Endotoxins. Infect. Immun..

[43] Lacaille-Dubois M.-A. (2019). Updated Insights into the Mechanism of Action and Clinical Profile of the Immunoadjuvant QS-21: A Review. Phytomedicine.

[44] Reinke S., Thakur A., Gartlan C., Bezbradica J.S., Milicic A. (2020). Inflammasome-Mediated Immunogenicity of Clinical and Experimental Vaccine Adjuvants. Vaccines.

[45] Marty-Roix R., Vladimer G.I., Pouliot K., Weng D., Buglione-Corbett R., West K., MacMicking J.D., Chee J.D., Wang S., Lu S. (2016). Identification of QS-21 as an Inflammasome-Activating Molecular Component of Saponin Adjuvants*. J. Biol. Chem..

[46] Talukdar P., Winegar P.H., Hudson G.A., Astolfi M.C.T., Inman J.L., Keasling J.D., Mukundan H. (2025). From Bark to Bench: Innovations in QS-21 Adjuvant Characterization and Manufacturing. Front. Immunol..

[47] Lal H., Cunningham A.L., Godeaux O., Chlibek R., Diez-Domingo J., Hwang S.-J., Levin M.J., McElhaney J.E., Poder A., Puig-Barberà J. (2015). Efficacy of an Adjuvanted Herpes Zoster Subunit Vaccine in Older Adults. N. Engl. J. Med..

[48] (2012). The RTS,S Clinical Trials Partnership. A Phase 3 Trial of RTS,S/AS01 Malaria Vaccine in African Infants. N. Engl. J. Med..

[49] Heath P.T., Galiza E.P., Baxter D.N., Boffito M., Browne D., Burns F., Chadwick D.R., Clark R., Cosgrove C., Galloway J. (2021). Safety and Efficacy of NVX-CoV2373 Covid-19 Vaccine. N. Engl. J. Med..

[50] Garcia L., Paradell H., Mouriño M., Alberca B., Urniza A., Vila A., Tarrats M., Plana-Durán J. (2011). Efficacy of an Inactivated and Adjuvanted “ZULVAC^®^ 8 OVIS” Vaccine Produced Using Single-Use Bioreactors. BMC Proc..

[51] Zulvac SBV | European Medicines Agency (EMA) https://www.ema.europa.eu/en/medicines/veterinary/EPAR/zulvac-sbv.

[52] Kenubih A. (2021). Foot and Mouth Disease Vaccine Development and Challenges in Inducing Long-Lasting Immunity: Trends and Current Perspectives. Vet. Med..

[53] Woldemariyam F.T., Negessu D., Bilata T., Muluneh A., Gebreweld D.S., Ebisa I.T., Paeshuyse J. (2023). Humoral Immune Response in Calves Vaccinated with Monovalent Vaccines or a Trivalent Combination Thereof and Matching of These Vaccines to the Selected Circulating Foot-and-Mouth Disease Viruses in Ethiopia. Vaccines.

[54] Huang W.-C., Zhou S., He X., Chiem K., Mabrouk M.T., Nissly R.H., Bird I.M., Strauss M., Sambhara S., Ortega J. (2020). SARS-CoV-2 RBD Neutralizing Antibody Induction Is Enhanced by Particulate Vaccination. Adv. Mater..

[55] Nyagwange J., Nene V., Mwalimu S., Henson S., Steinaa L., Nzau B., Tijhaar E., Pelle R. (2018). Antibodies to in Silico Selected GPI-Anchored *Theileria Parva* Proteins Neutralize Sporozoite Infection in Vitro. Vet. Immunol. Immunopathol..

[56] Nooraei S., Sarkar Lotfabadi A., Akbarzadehmoallemkolaei M., Rezaei N. (2023). Immunogenicity of Different Types of Adjuvants and Nano-Adjuvants in Veterinary Vaccines: A Comprehensive Review. Vaccines.

[57] Bessell P.R., Salmon G., Schnier C., Tjasink K., Al-Riyami L., Peters A. (2023). A High Level Estimation of the Net Economic Benefits to Small-Scale Livestock Producers Arising from Animal Health Product Distribution Initiatives. Front. Vet. Sci..

[58] Metwally S., Viljoen G., El Idrissi A. (2022). Veterinary Vaccines: Principles and Applications.

