# Supplementary material for: Immunogenicity of Theileria parva p67C Antigen Delivered via Adjuvanted CoPoP Liposomes in Cattle and Mice
Source: Vaccines (Basel). 2026 May 20;14(5):459. doi: 10.3390/vaccines14050459 (PMC13211421; doi:10.3390/vaccines14050459)
Supplement: Supplementary file 1 [file vaccines-14-00459-s001.zip › vaccines-4180496-supplementary.pdf]

Supplementary Material

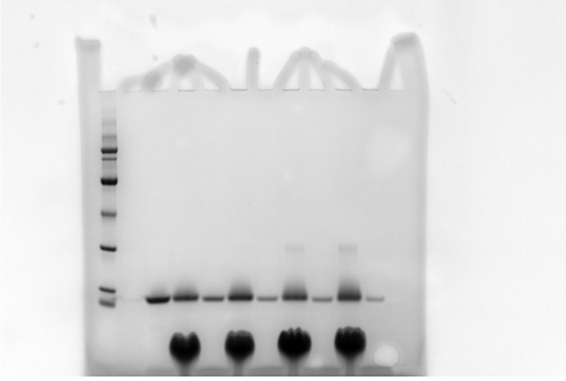

Figure S1. Unmodified Figure 1B. SDS-PAGE showing the binding capacity of p67C antigen to CoPoP liposomes after 3 hours of incubation in the dark, compared to soluble p67C (PBS) and assessed by SDS-PAGE.

Table S1: SDS-PAGE band intensities of p67C after binding assay: CA (CoPoP only), CQ (CoPoP-QS21), CP (CoPoP-PHAD), and CPQ (CoPoP-PHAD-QS21).

Lane	Sample name	Adjusted volume intensity
**1**	PBS-Sup	ND
**2**	PBS-Beads	28,491,864.47
**3**	CA-Sup	25,206,561.82
**4**	CA-Beads	13,650,573.45
**5**	CQ-Sup	29,676,330.57
**6**	CQ-Beads	7,203,946.76
**7**	CP-Sup	25,563,505.17
**8**	CP-Beads	6,590,421.05
**9**	CPQ-Sup	28,554,868.89
**10**	CPQ-Beads	3,322,632.75

ND states for Non-detected

Table S2: Components of CoPoP liposomes formulations used in the cattle experiment.

Liposome names	Liposome components	Concentration of components (mg/mL)
**CoPoP (CA)**	CoPoP	1
	DOPC	20
	CHOL	5
**CoPoP + QS21 (CP)**	CoPoP	1
	QS-21	0.4
	DOPC	20
	CHOL	5
**CoPoP + PHAD (CQ)**	CoPoP	1
	PHAD	0.4
	DOPC	20
	CHOL	5
**CoPoP + PHAD + QS21 (CPQ)**	CoPoP	1
	PHAD	0.4
	QS-21	0.4
	DOPC	20
	CHOL	5
